# Refractive Error and Risk of Early or Late Age-Related Macular Degeneration: A Systematic Review and Meta-Analysis

**DOI:** 10.1371/journal.pone.0090897

**Published:** 2014-03-06

**Authors:** Ying Li, JiWen Wang, XiaoJing Zhong, Zhen Tian, Peipei Wu, Wenbo Zhao, Chenjin Jin

**Affiliations:** 1 State Key Laboratory of Ophthalmology, Zhongshan Ophthalmic Center, Sun Yat-sen University, Guangzhou, China; 2 Department of Neurosurgery and Pituitary Tumor Center, The First Affiliated Hospital, Sun Yat-sen University, Guangzhou, China; Massachusetts Eye & Ear Infirmary, Harvard Medical School, United States of America

## Abstract

**Objective:**

To summarize relevant evidence investigating the associations between refractive error and age-related macular degeneration (AMD).

**Design:**

Systematic review and meta-analysis.

**Methods:**

We searched Medline, Web of Science, and Cochrane databases as well as the reference lists of retrieved articles to identify studies that met the inclusion criteria. Extracted data were combined using a random-effects meta-analysis. Studies that were pertinent to our topic but did not meet the criteria for quantitative analysis were reported in a systematic review instead.

**Main outcome measures:**

Pooled odds ratios (ORs) and 95% confidence intervals (CIs) for the associations between refractive error (hyperopia, myopia, per-diopter increase in spherical equivalent [SE] toward hyperopia, per-millimeter increase in axial length [AL]) and AMD (early and late, prevalent and incident).

**Results:**

Fourteen studies comprising over 5800 patients were eligible. Significant associations were found between hyperopia, myopia, per-diopter increase in SE, per-millimeter increase in AL, and prevalent early AMD. The pooled ORs and 95% CIs were 1.13 (1.06–1.20), 0.75 (0.56–0.94), 1.10 (1.07–1.14), and 0.79 (0.73–0.85), respectively. The per-diopter increase in SE was also significantly associated with early AMD incidence (OR, 1.06; 95% CI, 1.02–1.10). However, no significant association was found between hyperopia or myopia and early AMD incidence. Furthermore, neither prevalent nor incident late AMD was associated with refractive error. Considerable heterogeneity was found among studies investigating the association between myopia and prevalent early AMD (*P* = 0.001, I^2^ = 72.2%). Geographic location might play a role; the heterogeneity became non-significant after stratifying these studies into Asian and non-Asian subgroups.

**Conclusion:**

Refractive error is associated with early AMD but not with late AMD. More large-scale longitudinal studies are needed to further investigate such associations.

## Introduction

Age-related macular degeneration (AMD), a devastating disease affecting the macula, is the leading cause worldwide of irreversible central vision loss in people over 50 years old [1], [2], [3]. As a major public health issue, the precise cause of AMD remains unknown. However, several environmental and genetic factors, as well as their interactions, have been recognized as risk factors for the development and exacerbation of AMD. Aging, smoking and positive family history increase the predisposition of an individual to develop AMD [4], [5]. A gene polymorphism of complement factor H is considered to be the most consistent genetic risk factor for AMD [6].

Apart from these risk factors, refractive error, especially hyperopia, has also been considered to be associated with AMD in previous studies [9], [14]. Unlike AMD, refractive error is a common cause of correctible vision blur [7]. In either population-based or clinic-based studies [8], [9], a higher AMD prevalence has been found among hyperopic as compared with emmetropic eyes, though the strength of this association varies [10], [11], [12]. Some studies also reported a lower AMD risk in myopic eyes, while others failed to discover such a relationship [10], [13], [14], [15], [16], [17].

Prevention should be emphasized more strongly owing to the lack of effective treatments for AMD at present. It is therefore necessary to identify risk factors in an effort to prevent the development of AMD. We conducted this systematic review and meta-analysis based on published literature to provide evidence of the association between AMD and refractive error, including hyperopia, myopia, and per-diopter increase in spherical equivalent (SE). Axial length (AL), a parameter potentially relevant to refractive error, was also investigated.

## Methods

### (1) Search Strategy

We searched the Medline, Web of Science, and Cochrane Library databases to identify all potentially relevant published studies by using the following combination of terms with no restrictions (MeSH terms were not used to search the Web of Science): ((ARM or AMD or ARMD or “age-related macular degeneration” or “age-related maculopathy” or CNV or drusen or “macular degeneration”[MeSH] or “choroidal neovascularization”[MeSH] or “retinal drusen”[MeSH] or “geographic atrophy”[MeSH]) AND (“ocular parameter” or “axial length” or hyperop* or myop* or ammetropia or “refractive error” or hyperopia [MeSH] or myopia [MeSH]) AND (population or clinic or prospective or trial or control or “cross-sectional” or “case-control study”) NOT (animals[MeSH] not (animal[MeSH] and humans[MeSH])). The last search update was performed on July 27, 2013. The references cited in retrieved articles were also scanned for any additional relevant studies. Two authors independently conducted the search; any disagreements were solved by consensus. The current meta-analysis was conducted according to the guidelines in the Preferred Reporting Items for Systematic Reviews and Meta-Analyses.

### (2) Inclusion and Exclusion Criteria

Studies were considered eligible if they met the following criteria: A. explored the associations between hyperopia, myopia, SE, AL and AMD; B. clearly reported the method used to assess AMD, which was limited to the Wisconsin Age-Related Maculopathy grading system or the International Classification and Grading system [18], [19]; C. reported an effect estimate such as an odds ratio (OR) with 95% confidence interval (CI) or provided the raw data for calculation; D. in the case of multiple publications sharing the same sample, the study that best addressed our topic was included. We excluded animal studies, non-English articles, and abstracts. When there were studies that were appropriate for our topic but did not meet the criteria for quantitative analysis, we included them in the systematic review and listed the reasons for exclusion separately.

### (3) Data Extraction and Quality Assessment

Extracted information from each eligible study included: first author, publication year, study name, country in which the study was performed, age range or years of follow-up for patients with AMD, sample size (AMD patients/total population), response rate or follow-up rate, diagnostic standards for AMD, AMD type, adjusted confounders, definitions of hyperopia and myopia, and the effect estimates with corresponding 95% CIs. If a study addressed all or some of the aspects we studied, we presented the results separately. The most completely adjusted estimate was extracted when several estimates were provided. For studies reporting ORs stratified by gender or AMD subtype, we tried contacted the author to obtain the data without stratification; otherwise, we pooled the ORs to get an overall estimate [20]. Early or late AMD was considered the outcome measure in our study. When only the presence of any AMD was reported, the condition was classified as early AMD because of the low prevalence and incidence of late AMD. Study quality was ranked as high, moderate, or low (score categories 7–9, 4–6, 3–0, respectively) by using the Newcastle-Ottawa Quality Assessment Scale (NOS) [21]. Two independent reviewers conducted the data extraction and quality assessment, and solved disagreements by discussion.

### (4) Statistical Analysis

All statistical analyses were performed using STATA software (version 12.0, Stata Corporation, College Station, Texas). The fully adjusted, study-specific ORs were combined using a random-effects model, which accounts for both within- and between-study heterogeneities [22]. Pooled estimates were calculated according to the patient’s refractive error (hyperopia, myopia, per-diopter increase in SE, per-millimeter increase in AL) and the prevalence or incidence of early or late AMD. To explore the contribution of an individual study to the pooled estimates and between-study heterogeneity, sensitivity analysis was performed as well. Subgroup analysis was performed to explore possible sources of any observed heterogeneity.

We used Q and I^2^ statistics to assess the presence and amount of between-study statistical heterogeneity. *P*<0.1 was used as the cut-off for significant heterogeneity. I^2^ values of ≤24%, 25–49%, 50–74%, and ≥75% denoted no, low, moderate, and high heterogeneity, respectively [23]. Publication bias was estimated using Egger’s linear regression test and Begg’s test [24], [25]. A funnel test was used for the graphic display of these results. The standard error of log(OR) for each study was plotted against its log(OR). A two-tailed *P* value <0.05 was considered to be statistically significant.

## Results

### (1) Literature Search

**Figure 1:**
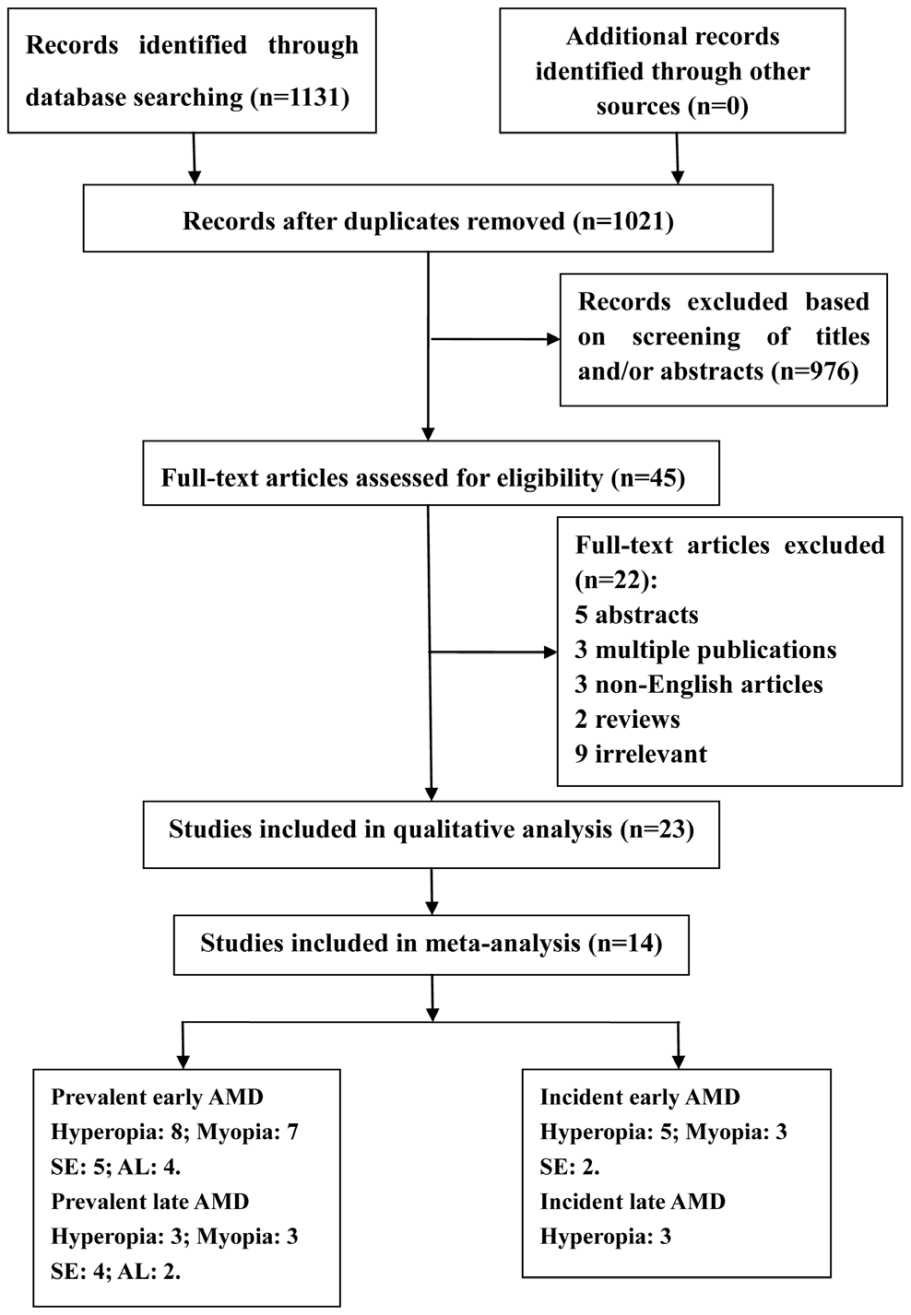
Flow chart for study selection: flow diagram showing the selection process for the inclusion of studies in the systematic review and meta-analysis. SE, spherical equivalent; AL, axial length.

A flow diagram of our literature search is presented in Figure 1. The search yielded 1131 articles: 523 from PubMed, 360 from the Web of Science, and 248 from the Cochrane library. After duplicate removal, 1021 titles and abstracts were assessed, of which 45 articles were found to be potentially relevant for inclusion and thus retrieved for full-text review. After a thorough review, 23 articles containing results pertinent to our topic were identified. Among these 23 articles, 14 population-based studies [9], [10], [11], [13], [14], [26], [27], [28], [29], [30], [31], [32], [33] met the criteria for meta-analysis, while the remaining 9 articles [8], [15], [17], [34], [36], [37], [38], [39], [40] were analyzed qualitatively and reported separately. Of the 14 included studies, 10 reported the association between refractive error and AMD prevalence, and 5 reported the association between refractive error and AMD incidence. We decided to qualitatively evaluate the remaining 9 articles [8], [15], [17], [34], [35], [36], [37], [38], [39] (5 case-control and 3 cross-sectional studies, including 2 articles from the Beijing Eye Study related to different aspects of our investigation) because most failed to provide data that could be used for a combined analysis or did not diagnose AMD according to standard guidelines.

### (2) Characteristics of Studies Included in the Meta-analysis

The 14 eligible studies included a total of 54091 individuals, 5814 of whom had AMD. The study characteristics are presented in Tables 1 and 2. All the extracted estimates are listed in Table 3. All studies reported age-adjusted ORs. In the Singapore Prospective Study Program (SPSP) [28] study, the results were stratified by gender without an overall estimate for refractive error and early AMD. Fortunately, we were able to obtain these data from the SPSP’s corresponding author. For the other studies with stratified results [10], [11], [27], [32], we pooled the stratified ORs to obtain an overall estimate. The Singapore Indian Eye Study (SIES) [26], France-DMLA study [14], and the Visual Impairment Project (VIP) [29] did not report the ORs for early AMD patients; therefore, ORs for any AMD were extracted. In most cases, refractive error was assessed automatically and subjectively. While the SPSP [28] measured refractive error automatically, the France-DMLA study [14], the Tromso Eye Study (TES) [35], and the VIP [29] did not report the method used to calculate refractive error. AL was detected by non-contact partial coherence laser interferometry in the Central India Eye and Medical Study (CIEMS) [13], SIES and Singapore Malay Eye Study (SiMES) [9], but by A-scan ultrasound in the Los Angeles Latino Eye Study (LALES). The defined cut-off points for myopia and hyperopia differed among studies (−0.5 diopters (D) vs. −1.0 D, 0.5 D vs. 1.0 D). In the DMLA study, TES and the Beijing Eye Study (BES) [30], the definition of hyperopia was not defined clearly. The TES only reported the per-diopter increase in SE in eyes with late AMD as compared to eyes without late AMD.

**Table 1:** Characteristics of the 10 studies included in meta-analysis of refractive errors and prevalent AMD.

Author Year	Study	Country	Age (years)	AMD/TotalNumbers	Response Rate (%)	Adjusted Factors	Cut-off of H,M,E	AMD Criteria	AMD Type	NOS Score
Jonas 2012 [13]	CIEMS	India	30–100	223/4542	80.1	Age	−0.5 D, 0.5 D	W	Early	8
Pan 2013 [26]	SIES	Singapore	40–84	202/3337	75.6	Age, sex, BMI, smoking, HT, education, cholesterol level	−0.5 D, 0.5 D	W	Any	9
Lavanya 2010 [9]	SiMES	Singapore	40–80	179/3070	78.7	Age, sex, height, smoking, education, systolic BP	−0.5 D, 0.5 D	W	Early,late	9
FraserBell 2010 [27]	LALES	USA	40–80	550/5875	75.4	Age, sex, smoking	−0.5 D, 0.5 D	W	Early,late	8
Wang 1998 [11]	BMES	Australia	>49	257/3351	82.4	Age, sex, smoking,family history	−1.0 D, 1.0 D	W	Early,late	9
Ikram 2003 [10]	RS	Netherlands	>55	536/6209	78.0	Age, sex, smoking, BP	−0.5 D, 0.5 D	I	Early,late	8
Chaine 1998 [14]	DMLA	France	50–89	1844/3688	96.7	Age, sex	Not mention	W	Any	7
Cheung 2012 [28]	SPSP	Singapore	40–95	211/3172	80.2	Age, sex, smoking	−1.0 D, 1.0 D	W	Early	8
McCarty 2001 [29]	VIP	Australia	40–98	686/4345	92	Age	1.0 D	I	Any	6
Erke 2012 [35]	TES	Norway	65–87	92/2631	87	Age, sex, self-reported cataract	Not mention	I	Late	7

Abbreviations: AMD, age-related macular degeneration; CIMES, Central India Eye and Medical Study; SIES, the Singapore Indian Eye Study; SiMES, the Singapore Malay Eye Study; LALES, Los Angeles Latino Eye Study; BMES, the Blue Mountains Eye Study; RS, the Rotterdam Study; SPSP, the Singapore Prospective Study Program; VIP, the Visual Impairment Project; TES, the Tromso Eye Study; BMI, body mass index; HT, hypertension; BP, blood pressure; D, diopter; H, hyperopia; M, myopia; E, emmetropia; W, Wisconsin Age-related Maculopathy Grading System; I, International Classification and Grading system; SE, spherical equivalent; AL, axial length; NOS, Newcastle-Ottawa scale.

**Table 2:** Characteristics of 5 studies included in meta-analysis of refractive errors and incident AMD.

Author Year	Study	Country	AMD/TotalNumbers	Follow-upYears	Follow-upRate(%)	Adjusted Factors	Cut-off of H,M,E	AMD Criteria	AMD Type	NOS Score
Wang 2004 [32]	BMES	Australia	236/2335	5	75.1	Age, sex, smoking	−1.0 D, 1.0 D	W	Early, late	8
Wong 2002 [31]	BDES	USA	239/3306	10	56.1	Age	−0.75 D, 0.75 D	W	Early, late	8
You 2012 [30]	BES	Beijing	146/3049	5	73.2	Age, body height, education, profession and smoking	Not mentioned	I	Early	6
Buch 2005 [33]	CCES	Copenhagen	146/359	14	31.8	Age, sex	1.0 D	W	Early, late	6
Ikram 2003 [10]	RS	Netherlands	497/4822	5	72.8	Age, sex, smoking, blood pressure, atherosclerosis	−0.5 D, 0.5 D	I	Any	8

Abbreviations: AMD, age-related macular degeneration; BMES, the Blue Mountain Study; BDES, the Beaver Dam Eye Study; BES, the Beijing Eye Study; CCES, the Copenhagen City Eye Study; RS, the Rotterdam Study; D, diopter; H, hyperopia; M, myopia; E, emmetropia; W, Wisconsin Age-related Maculopathy Grading System; I, International Classification and Grading system; SE, spherical equivalent; AL, axial length; NOS, Newcastle-Ottawa scale.

**Table 3:** ORs and 95% CIs of the associations between AMD and refractive errors.

Authors	Early AMD				Late AMD			
	Hyperopia	Myopia	SE	AL	Hyperopia	Myopia	SE	AL
**Prevalent AMD**								
Jonas [13]	–	–	1.15(1.06,1.25)	0.81(0.69,0.95)	–	–	–	–
Pan [26]	0.84(0.56,1.25)	0.45(0.25,0.79)	1.14(1.02,1.28)	0.76(0.65,0.89)	–	–	–	–
Lavanya [9]	1.13(1.11,1.15)	0.74(0.47,1.15)	1.08(1.01,1.16)	0.78(0.64, 0.94)	–	–	1.06(0.87,1.30)	1.22(0.71,2.08)
FraserBell [27]	1.03(0.78,1.13)	0.98(0.81.1.14)	–	0.81(0.70, 0.92)	1.00(0.40,2.40)	0.60(0.10,1.90)	–	0.90(0.60,1.30)
Wang [11]	1.09(0.77,1.41)	0.62(0.25,0.99)	1.14(1.04,1.25)	–	0.55(0.27,1.34)	0.80(0.26,1.34)	0.99(0.86,1.13)	–
Ikram [10]	1.37(0.92,2.05)	0.97(0.65,1.60)	1.09(1.04,1.14)	–	0.87(0.47,1.59)	0.71(0.31,1.64)	1.09(1.00,1.19)	–
Chaine [14]	1.33(1.11,1.59)	0.99(0.78,1.25)	–	–	–	–	–	–
Cheung [28]	1.04(0.72,1.50)	0.53(0.37,0.78)	–	–	–	–	–	–
McCarty [29]	1.23(1.02,1.48)	–	–	–	–	–	–	–
Erke [35]	–	–	–	–	0.83(0.76,0.90)	–	–	–
**Incident AMD**								
Wang [32]	0.83(0.60,1.06)	0.64(0.28, 1.00)	1.10(0.98,1.15)	–	0.97(0.23,1.72)	–	1.10(0.90,1.20)	–
Wong [31]	0.90(0.70,1.10)	1.00(0.70,1.30)	–	–	1.20(0.60,2.30)	–	–	–
You [30]	1.15(1.00,1.33)	–	–	–	–	–	–	–
Buch [33]	0.90(0.60,1.60)	–	–	–	1.10(0.60,2.10)	–	–	–
Ikram [10]	1.13(0.88,1.38)	0.74(0.51,1.00)	1.05(1.01, 1.10)	–	–	–	–	–

Abbreviations: AMD, age-related macular degeneration; SE, spherical equivalent; AL, axial length; OR, odds ratio; CI, confidence interval.

Among the studies investigating refractive error and AMD incidence, all reported the reasons for exclusion from the follow-up analysis. The duration of follow-up ranged from 5–14 years. The Copenhagen City Eye Study (CCES) [33] had the lowest follow-up rate (31.8%), perhaps owing to the long follow-up period of 14 years. The Rotterdam Study [10] reported the association between refractive error and AMD incidence without a separate report for early AMD. Only the Blue Mountain Eye Study [32] reported the association between SE and the incidence of late AMD. Only the Beaver Dam Eye Study [31] reported the association between myopia and late AMD incidence. These aspects of the investigation were therefore not included as part of the meta-analysis.

The scores awarded for study quality varied. The quality of 10 studies ranked as high, and the remaining 3 studies were ranked as moderate. No attempt was made to weight studies based on the quality score to avoid introducing subjective bias to the meta-analysis.

### (3) Hyperopia and AMD

**Figure 2:**
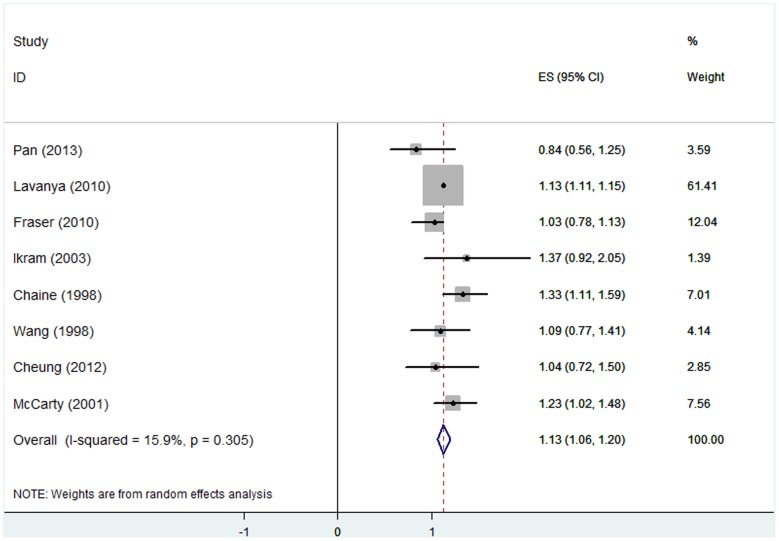
Hyperopia and prevalent early AMD: forest plot of pooled odds ratios for the random-effects meta-analysis of early AMD prevalence and hyperopia. CI, confidence interval.

**Figure 3:**
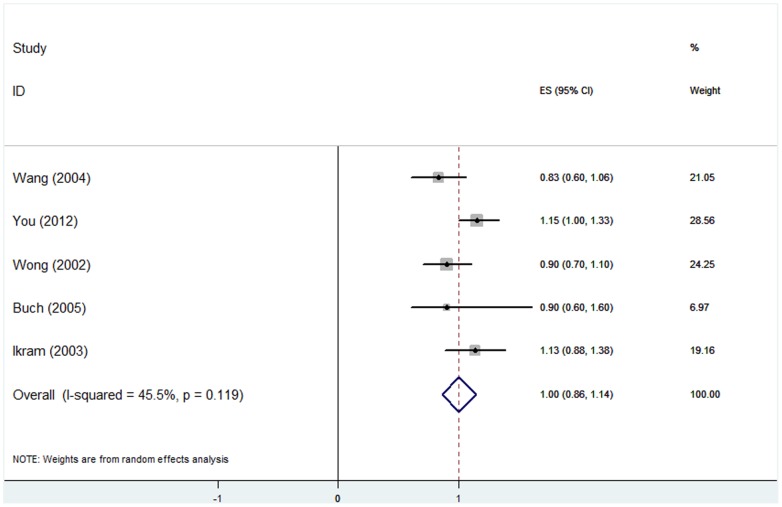
Hyperopia and early AMD incidence: forest plot of pooled odds ratios for the random-effects meta-analysis of early AMD incidence and hyperopia. CI, confidence interval.

All the pooled estimates of associations between AMD and refractive error are summarized in Table 4. Eight studies [9], [10], [11], [14], [26], [27], [28], [29] involving 33047 individuals had investigated the association between hyperopia and early AMD prevalence. Five studies [10], [30], [31], [32], [33] that included 13871 individuals reported the association between hyperopia and early AMD incidence. The adjusted OR for each study and the overall estimates are presented in Figures 2 and 3. A random-effects model showed that the association between hyperopia and early AMD prevalence was statistically significant. This analysis yielded a pooled OR of 1.13 (95% CI, 1.06–1.20). The results from longitudinal studies were inconsistent. The pooled results showed that hyperopic eyes had nearly the same incidence of early AMD as emmetropic eyes (OR, 1.00; 95% CI, 0.86–1.14). For hyperopia and late AMD, the associations were non-significant; the ORs and 95% CIs for AMD prevalence and incidence were 0.76 (0.38–1.14) and 1.08 (0.63–1.53), respectively. There was no statistically significant heterogeneity between studies. The stability and reliability of the pooled estimates were confirmed by leave-one-out sensitive analysis, which suggested that the influence of any individual data set on the pooled OR was not significant. There was no evidence of possible publication bias as indicated by non-significance using Egger’s and Begg’s tests (all *P*>0.05). Subgroup analysis based on geographic context revealed that the relationship of AMD and hyperopia was similar in Asian and non-Asian countries.

**Table 4:** Pooled estimates of meta-analysis for refractive errors and AMD.

	Prevalent			Incident			Subgroups of prevalent AMD						
							Asia			Non-Asia			
	OR(95% CI)	*P*	I^2^(%)	OR(95% CI)	*P*	I^2^(%)	OR(95% CI)	*P*	I^2^(%)	OR(95% CI)	*P*	I^2^(%)	
**Early AMD**													
**Hyperopia**	1.13 (1.06, 1.20)	0.31	15.9	1.00 (0.86–1.14)	0.12	45.5	1.12 (1.05, 1.19)	0.34	11.3	1.16 (0.97, 1.36)	0.14	49.7	
**Myopia**	0.75 (0.56, 0.94)	0.00	72.2	0.80 (0.60–1.00)	0.26	25.6	0.56 (0.42, 0.69)	0.60	0	0.98 (0,85, 1.11)	1.00	0	
**SE**	1.10 (1.07, 1.14)	0.75	0	1.06 (1.02, 1.10)	0.31	3.7	_	–	–	–	–	–	
**AL**	0.79 (0.73, 0.85)	0.93	0	–	–	–	–	–	–	–	–	–	
**Late AMD**													
**Hyperopia**	0.76 (0.38, 1.14)	0.66	0	1.08 (0.63, 1.53)	0.92	0	–	–	–	–	–	–	
**Myopia**	0.73 (0.35, 1.11)	0.93	0	–	–	–	–	–	–	–	–	–	
**SE**	0.98 (0.83, 1.13)	0	85.6	–	–	–	–	–	–	–	–	–	
**AL**	0.97 (0.65, 1.28)	0.42	0	–	–	–	–	–	–	–	–	–	

Abbreviations: AMD, age-related macular degeneration; CI, confidence interval; OR, odds ratio; SE, spherical equivalent; AL, axial length.

### (4) Myopia and AMD

**Figure 4:**
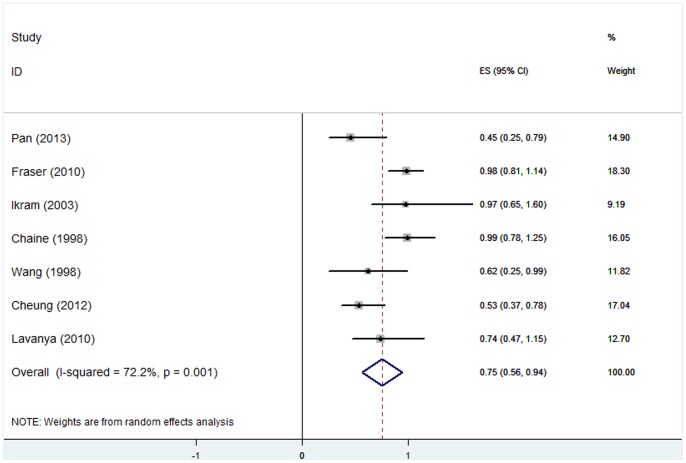
Myopia and early AMD prevalence: forest plot of pooled odds ratios for a random-effects meta-analysis of early AMD prevalence and myopia. CI, confidence interval.

**Figure 5:**
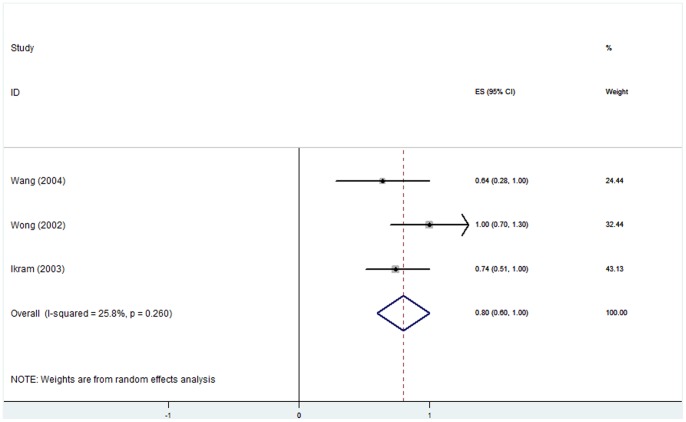
Myopia and early AMD incidence: forest plot of pooled odds ratios for a random-effects meta-analysis of early AMD incidence and myopia. CI, confidence interval.

**Figure 6:**
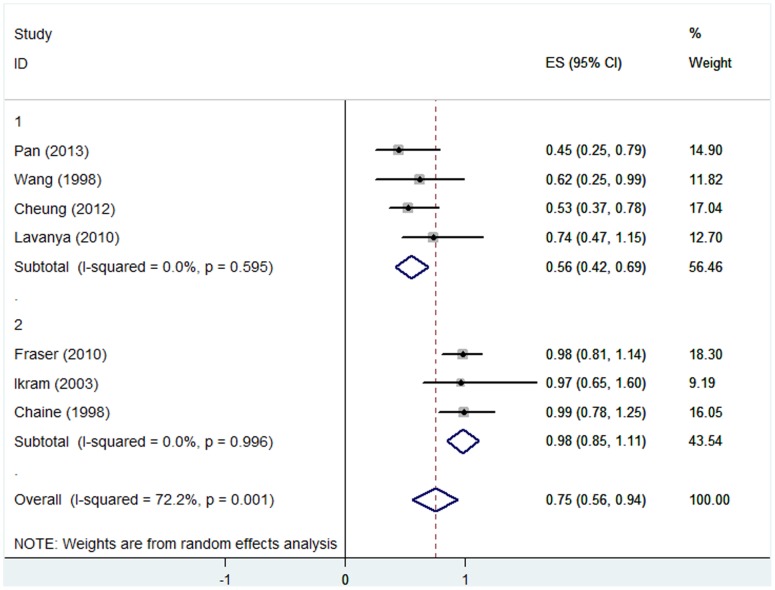
Myopia subgroups and early AMD prevalence: forest plot of risk estimates for early AMD prevalence and myopia stratified by geographic location (Asia or non-Asia subgroups). CI, confidence interval.

The pooled ORs for myopia [10], [11], [14], [26], [27], [28] and early AMD prevalence, early AMD incidence, and late AMD prevalence were 0.79 (95%CI, 0.61–0.97), 0.80 (95% CI, 0.60–1.00), and 0.73 (95%CI, 0.63–1.53), respectively. The results for early AMD are shown in Figures 4 and 5. Significant heterogeneity existed among studies reporting on the association between myopia and early AMD prevalence (*P* = 0.001, I^2^ = 72.2%). In the sensitivity study, no individual study was found to significantly affect the pooled estimate. To explore the potential source of heterogeneity in the relationship between myopia and early AMD prevalence, we performed a subgroup study. The heterogeneity disappeared when we stratified the studies by geographic location. For studies performed in Asia, the tests for heterogeneity yielded I^2^ = 0.0%, *P* = 0.60, and for those in non-Asian countries, the results were I^2^ = 0.0%, *P* = 1.00. The respective ORs were 0.56 (95%CI, 0.42–0.69) and 0.98 (0.85–1.11) (Figure 6).

### (5) SE, AL and AMD

The per-diopter increase toward hyperopia in SE was associated with early AMD prevalence as well as incidence, with the pooled estimates being 1.10 (1.07–1.13) and 1.06 (1.02–1.10). However, for late AMD prevalence, the pooled estimate was 0.98 (0.83–1.13). The per-millimeter increase in AL was inversely associated with the prevalence of early AMD, with the pooled estimate being 0.79 (0.73–0.85). Similarly, late AMD prevalence was not associated with increased AL (OR, 0.97; 95%CI, 0.65–1.28). Significant heterogeneity was found in studies investigating SE and late AMD prevalence (*P* = 0.00, I^2^ = 85.6). After removing the TES study, no heterogeneity existed (*P* = 0.49, I^2^ = 0.0). No publication bias was found, with all P-values for results on the Egger’s and Begg’s tests >0.5.

### (6) Systematic Review for Refractive Error and AMD in Studies Excluded from the Meta-analysis

Table S1 lists the characteristics of all systematically reviewed articles and reasons for excluding them from the meta-analysis. Nine articles [8], [15], [17], [34], [35], [36], [37], [38], [39], [40] derived from 8 studies (2 separate articles [17], [37] for the Beijing Eye Study), including 3 cross-sectional and 5 case-control studies, investigated our topic but were not appropriate for a meta-analysis. Goldberg et al [15] diagnosed AMD using the criteria developed by the National Eye Institute, which consider visual acuity in diagnosis. The authors reported that the risk of AMD was increased in hyperopic eyes as compared with emmetropic eyes (OR, 1.61; 95%CI, 1.15–2.25) in a population of about 10,000 people. In the Beijing Eye Study, it was found that hyperopic refractive error was the single most important risk factor for AMD other than age in Chinese individuals over 40 years old (*P = *0.008, 95%CI; 1.04–1.28) [17]. The study also reported significantly lower frequencies of early macular degeneration (OR, 3.0; 95%CI, 1.25–7.51) and late macular degeneration (*P*<0.001; OR, 6.33) in high myopes as opposed to those without high myopia [37]. Ulvik et al [38] investigated 663 persons aged over 65 years in Norway, and found no statistically significant relationship between AL (*P = *0.55) or refraction (*P = *0.29) and AMD. Tao et al [36] compared 379 exudative AMD patients with 191 controls in a hospital in Germany. The results showed that the AMD group had significantly shorter AL (23.31±0.75 vs. 24.20±1.56 mm; *P*<0.001) and was more hyperopic (0.65±2.14 vs. −1.71±4.57 D; *P*<0.001). The Eye Disease Case-Control Group [39] considered visual acuity <6/6 or distortion on the Amsler grid as a part of the diagnostic criteria for AMD. They reported that compared to emmetropes, individuals with hyperopia were more likely to have neovascular AMD with an OR of 1.7 (1.1–2.6). In the Age-Related Eye Disease Study [34], 4519 persons aged 60–80 years were stratified into four groups (intermediate drusen, large drusen, geographic atrophy, neovascular degeneration). Hyperopia was associated with large drusen (OR, 1.28; 95%CI, 1.04–1.57) and neovascular degeneration (OR, 2.31; 95%CI, 1.67–3.21). Sandberg et al [8] investigated the association between hyperopia and pre-diagnosed neovascular AMD in a case-control study and found that patients with a refractive error of +0.75 D or greater were more likely to have neovascularization than patients with other types of refractive error (OR, 2.40; 95%CI, 1.53–3.78). In another study performed by Boker et al [40], the general German population was set as the control group. The authors reported that an eye with a refractive error of +3 D was 6.2 times more likely to develop choroidal neovascularization than an emmetropic eye.

## Discussion

The identification of risk factors that could easily be assessed by physicians to screen for individuals at high risk before irreversible visual loss would improve public health and decrease associated economic costs. We therefore performed the current systematic review and meta-analysis to assess the association between refractive error and AMD by summarizing all available published evidence.

The current meta-analysis investigates the relationships between hyperopia, myopia, per-diopter increase in SE, per-millimeter increase in AL and the prevalence or incidence of AMD. Compared with emmetropic eyes, hyperopic eyes had a 13% higher risk of early AMD. In contrast, myopic eyes had a 25% lower risk of early AMD. Furthermore, per-diopter increases toward hyperopia were associated with a 10% increase in early AMD prevalence and a 6% increase in early AMD incidence. Each millimeter-increase in AL was associated with a 21% reduction in odds. However, no evidence indicated that refractive error was associated with the prevalence or incidence of late AMD. Significant between-study heterogeneity was observed for myopia and early AMD prevalence as well as for SE and late AMD prevalence. Subgroup analysis suggested that geographic location might be the main origin of the heterogeneity observed for myopia and early AMD. The TES study might represent the source of heterogeneity in SE and late AMD prevalence, since the control group in this study was eyes without late AMD rather than normal eyes. It seemed that the protective effect of myopia was stronger in studies performed in Asian countries compared to those performed in non-Asian countries (OR, 0.58 vs. 0.98). The higher prevalence of myopia in Asia might have influenced our results. However, caution should be undertaken when explaining this result because of the small number of studies included in each subgroup. Publication bias describes the lower tendency for studies reporting uninteresting or negative results to go published, which affects the reliability and validity of the results of a meta-analysis. Using a funnel plot combined with Egger’s linear regression test and Begg’s test, publication bias can be measured statistically. No evidence of publication bias was found in our meta-analysis. Notably, this type of publication bias analysis has limited value given that a considerable number of studies were reviewed systematically.

During the past few decades, although efforts have been made to explore the reasons for the association between AMD and refractive error, the precise mechanisms remain unclear. Previous studies have found that shorter hyperopic eyes are likely to have increased scleral rigidity, which results in increased choroidal vascular resistance and consequently reduces the transfer of oxygen and nutrients to the outer retina and finally impairs retinal pigment epithelium function. A hemodynamic model for the pathogenesis of AMD established by Friedman et al [41] demonstrated that the coefficient of scleral rigidity was inversely proportional to AL. Degeneration was closely linked to scleral compliance. It is therefore postulated that hyperopic eyes with naturally shorter AL might suffer metabolic problems and be at greater risk of developing AMD. Pallikaris et al [42] found that patients with neovascular AMD had increased ocular rigidity as compared with non-neovascular AMD and control patients. It has also been shown that choroidal resistance was higher in documented AMD cases as compared to age- and sex-adjusted controls [43]. In addition, the relative hypoxic conditions in hyperopic eyes may cause increased cytokine production. Jonas et al [44] reported that the intraocular concentration of vascular endothelial growth factor (VEGF) decreased significantly with increasing myopia, and as a corollary, with increasing AL. The larger ocular volume in myopic eyes was thought to dilute intraocular VEGF, leading to decreased angiogenesis. The findings in our meta-analysis coincide with these mechanisms.

Meta-analysis, an important statistical method for revealing trends that might not be apparent in a single study, increases the reliability of results by pooling independent but similar studies. The strengths of our meta-analysis include the population-based design of all the included studies and the substantial number of individuals studied. These factors minimize selection bias and increase the statistical power of the analysis significantly. Second, because the methods used to diagnose AMD differed among the included studies, we made efforts to maximize clinical homogeneity by excluding studies that did not use the standard criteria for diagnosing AMD [15], [39] from the mete-analysis. Before the Wisconsin Age-Related Maculopathy grading system and International Classification and Grading system were established, the diagnosis of AMD differed widely among studies. In some studies, the level of visual acuity was considered in diagnosis, which is no longer the case in general practice. This could have resulted in the over- or underestimation of AMD prevalence or incidence and subsequently biased the reported effect estimates. Third, though the associations between hyperopia, myopia and early AMD prevalence were weak (95% CIs, 1.06–1.20 and 0.56–0.94), the significant associations between per-D increases in SE, per-millimeter increases in AL and early AMD prevalence reinforced the confidence level of the pooled results. Fourth, we comprehensively analyzed the relationship between AMD (early/late, prevalence/incidence) and refractive error (hyperopia, myopia, SE, and AL). Finally, during the process of peer review of submission to a different journal, a similar article was published [45]. The association between refractive error and AMD was reported in the article, but AMD was not classified as early or late. Apart from the identification of an association between refractive error and early AMD, we found that no association existed between refractive error and late AMD. Furthermore, we included more studies than prior reports and qualitatively reviewed the excluded articles that were nonetheless highly related.

It is necessary to emphasize the limitations of our meta-analysis for proper interpretation of the results. First, only English-language articles were included, which limited the resources included in our meta-analysis. The number of studies in the systematic review was larger than that in the meta-analysis for the prevalence of early AMD. Among those included in the systematic review, the relationships between refractive error and AMD were inconsistent. The exclusion of these studies increased the clinical homogeneity of our study, but also introduced bias to the pooled results. Second, the relatively small number of included studies made it difficult to perform more detailed subgroup analyses. Third, the level of confounders adjusted for varied among studies included in the meta-analysis. Inadequate confounder control may have biased the results. All included studies adjusted for age, the most accepted risk factor for AMD. However, other common risk factors such as sex, smoking history, cardiovascular disease, and educational levels were not balanced across all studies. Fourth, the duration of follow-up varied largely among studies investigating the relationship between AMD incidence and refractive error. The reasons for loss to follow-up were not fully reported. Finally, we did not attempt to investigate different levels of refractive error (low, moderate, and high myopia or hyperopia) in relation to different subtypes or signs of AMD (geographic or neovascular AMD, drusen or pigmentation abnormalities). Several studies previously identified such associations. The LALES reported a protective effect of longer AL against the development of soft, indistinct drusen and retinal pigmentation. The risk for neovascular AMD was also reported to be higher with increasing degrees of hyperopia [8], [40]. Nevertheless, owing to the paucity of relative studies and the heterogeneity of published reports, we could not perform a meta-analysis based on these results.

In conclusion, the results of this meta-analysis indicate that hyperopia and increasing diopters are associated with an increased risk of early AMD. An inverse association existed between prevalent early AMD and myopia or increased AL. These associations might be taken into account to manage disease risk and establish a routine eye examination program, although such associations need to be evaluated further because of the negative relationships among hyperopia, myopia, and incident AMD. There is a need for more high-quality studies to assess the associations longitudinally in order to better clarify the role of refractive error in the development and progression of AMD. Studies are also warranted to elucidate the pathophysiologic mechanisms underlying the associations.

## Supporting Information

Table S1 **Characteristics and reasons for exclusion of studies excluded from meta-analysis.**
(PDF)

Checklist S1 **PRISMA checklist.**
(DOC)
