# Supplementary material for: Refractive Error and Risk of Early or Late Age-Related Macular Degeneration: A Systematic Review and Meta-Analysis
Source: PLoS One. 2014 Mar 6;9(3):e90897. doi: 10.1371/journal.pone.0090897 (PMC3946285; doi:10.1371/journal.pone.0090897)
Supplement: Table S1 — Characteristics and reasons for exclusion of studies excluded from meta-analysis. (PDF) [file pone.0090897.s001.pdf]

Table S1. Characteristics and reasons for exclusion of studies excluded from meta-analysis refractive error and AMD

Author year	Study(design)	Results	Reasons for exclusion
Anand, 2000[34]	Population-based case-control study including 4519 persons aged 60-80 years and stratified into four case groups (intermediate drusen, large drusen, GA and NV) and one control group.	Statistically significant association existed between large drusen, NV-AMD and hyperopia (OR, 1.28; 2.31 respectively) in multivariable analysis	Only reporting stratified ORs and ORs for intermediate drusen and GA group were not available.
Tao, 2010[36]	Clinical case-control study included 379 neovascular AMD treated by anti-VEGF drugs and 191 controls underwent surgery of catarate in Germany.	The AMD group was significantly shorter in AL compared with control group (23.31±0.75 vs 24.20±1.56mm, P<0.001), and was significantly more hyperopic (0.56±2.14 vs -1.74±4.57 D, P<0.001)	Reporting the refractive errors as continuous values without ORs. Moreover, it only studied exudative AMD.
Xu, 2006[17] 2007[37]	Population-based cross-sectional study (the Beijing Eye Study) included 4319 adult Chinese older than 40 years.	Compared with non-highly myopic group, the highly myopic group had a smaller mean size of macular drusen, and lower frequencies of early or late AMD (P<0.001).<br>Early ARM was statistically associated with hyperopic refractive error (P=0.008, 95% CI, 1.04-1.28)	Compared between highly and non-highly myopic groups.No relative OR reported.
Ulvik, 2005[38]	Population based prevalence study that included 663 persons aged over 65 years.	No statistically significant association between AL/refraction and AMD.	Reporting the refractive errors as continuous values
Goldberg 1988[15]	Population-based cross-sectional study (the First National Health and Nutrition Examination Survey) in USA included persons at least 45 year old.	ORs for the association between hyperopia (>1.0D), myopia (<-1.0D) and AMD were 1.43 (1.04-1.97), 1.28 (0.67-2.43) respectively compared with emmetropia.	Did not use the standardized criteria to diagnose AMD.

Table S1(continued)

Author year	Study(design)	Results	Reasons for exclusion
Boker, 1993[40]	Clinical case-control study including 186 eyes with NV-AMD compared with the general population of German.	It was found that the risk for the development of NV-AMD increased with increasing degrees of hyperopia. An eye with a refractive error of +3D was 6.2 times more likely to develop CNV than was an emmetropic eye.	No clear statement of AMD diagnosis and the control group was the general population.
Sandberg, 1993[8]	Clinic-based case-control study with 198 unilateral NV-AMD patients and 129 controls with bilateral dry disease.	Patients with the unilateral NV form had an average spherical equivalent that was 1.0 D more hyperopic than that of controls ($P < 0.001$). Patients with a refractive error of +0.75 D or greater were more likely to have the NV-AMD compared with patients with other refractive errors (OR, 2.40; 95% CI, 1.53-3.78; $P < 0.001$).	No clear statement of method for AMD diagnosis and the control group was the patients with refractive error less than +0.75D. Only NV-AMD was studied.
The Eye Disease Case-control Group, 1992[39]	Multi-center based case-control study consisting of 421 patients with NV-AMD and 615 controls aged between 55-80 years old.	ORs for the association between hyperopia (>1.0D), emmetropia (-1.0D≤SE≤1.0D) and AMD were 1.5 (0.9-2.4), 1.1 (0.6-1.8) respectively compared with myopia (<-1.0D) in multivariate analysis.	Did not use the standardized criteria to diagnose AMD. Investigated refractive error and late AMD.

Abbreviations: AMD, age-related macular degeneration; VEGF, vascular endothelial growth factor; GA, geographic atrophy; NV, neovascular; D, dopter; CI, confidence interval; OR, odds ratio; SE, spherical equivalent; CNV, choroidal neovascularization.
